# The Anatomy of the Large American Fluke (*Fasciola Magna*), and a Comparison with Other Species of the Genus Fasciola, S. St.

**Published:** 1894-03

**Authors:** Charles Wardell Stiles

**Affiliations:** Zoölogist, Bureau of Animal Industry


THE JOURNAL

OF

COMPARATIVE MEDICINE AND

VETERINARY ARCHIVES.

VOL. XV. MARCH, 1894. NO. 3.

### THE ANATOMY OF THE LARGE AMERICAN FLUKE (*FASCIOLA MAGNA*), AND A COMPARISON WITH OTHER SPECIES OF THE GENUS FASCIOLA, S.ST.

BY CHARLES WARDELL STILES, PH.D.,
*Zoölogist, Bureau of Animal Industry.*

### CONTAINING ALSO A LIST OF THE CHIEF EPIZOÖTICS OF FASCIOLIASIS (DISTOMATOSIS) AND A BIBLIOGRAPHY OF *FASCIOLA HEPATICA*.

BY ALBERT HASSALL, M.R.C.V.S.

CONTENTS.

INTRODUCTION.
List of epizoötics (By A. Hassall).

I.—*Fasciola magna:*
Synonymy.
Hosts.
Literature.
Geographical distribution.
Historical review.
Morphology.
Specific diagnosis.

II.—*Fasciola hepatica:*
Synonymy.
Hosts.
Specific diagnosis.
Intermediate hosts.
Literature (By Albert Hassall).

III.—*Fasciola gigantea:*
Synonymy.
Hosts
Geographical distribution.
Literature.
Historical review,
Specific diagnosis.

IV.—*Fasciola Jacksoni:*
Synonymy.
Host.
Geographical distribution.
Literature.
Historical review.
Specific diagnosis.

V.—*The generic name FASCIOLA.*

VI.—*Conclusions.*

VII.—*Compendium of Fasciolas,*
Arranged according to the hosts.

### INTRODUCTION.

It has been known for many years that liver-flukes are found in a number of the domesticated animals, and that they at times cause a high rate of mortality on the farms, more especially among the sheep and cattle. The form which has heretofore attracted the most attention is the "common liver-fluke" (*Fasciola hepatica L.*), which has repeatedly caused serious damage to the sheep industry of various countries, as is shown by the following:

### List of the Chief Epizoötics of Fascioliasis (Distomatosis).

Compiled by Albert Hassall.

Unless otherwise stated the epizoötic was caused by *F. hepatica*. The data of the earlier outbreaks are necessarily very incomplete.

### 1500—1599.

'26. Cosimus observed an epizoötic of distomatosis in Holland (after Wolfgang Franzius), cited from Braun.

'42. Gentiles Arnulphus. Cited by Haller.

'47. Gabucinus.

'52. Cornelius Gemma refers to an epizoötic in Holland. Davaine states that this is the first epizoötic of distomatosis mentioned in history. Gemma speaks of the outbreak as "*lues infanda pecoris*" (unheard of pestilence of sheep).

### 1600—1699.

'63–'65. In the Duchy of Coburg, Fromann observed an epizoötic which attacked the sheep of all ages, the calves and heifers up to two years of age, but not the oxen or cows. The hares and deer on the plains and forests died of this disease. Horses, goats and hogs were not exempt. In four folds, containing more than 3,000 sheep, not over 40 remained. (Davaine.)

'64. Williéno Valentin. Island of Zealand. (Hurtrel D'Arboval). This could not be traced. See Willius '74.

'74. Willius observed an epizoötic in Seeland in which nearly all the cattle were affected (Davaine)

Braun gives this epizoötic as due to "Blasenwürmer" (bladderworms—Cysticercus, Cœnurus, Echinococcus).

### 1700—1799.

'35. Outbreak mentioned in Ellis' Shepherds' Sure Guide (1749), affecting the Vale of Aylesbury, England. Deer, sheep, lambs, hares and coneys (rabbits).

"The dead bodies of Rotten Sheep were so numerous that their carrion stench and smell proved extremely offensive to the neighboring parts and to passant travellers."

'43–'44. Nearly all the sheep in the territory of Arles were destroyed. (Davaine.)

'47. An epizoötic in Vale of Aylesbury similar to that of '35.

'61. All the flocks of Aveyron destroyed. (Davaine.)

61–'62. Great losses in sheep in northern part of France, especially in Bas Boulonnais. Very wet season. (Paulet, after Davaine.)

'66. Outbreaks of distomatosis all over England. (Reported by Mills in Treatise on Cattle, 1776.)

'92. Harrison (1804) mentions outbreak in England.

### 1800–1894.

'09. Terrific epizoötic in nearly all parts of France; in Beaujolais and Lyonnais, the entire herds of Merinos died. (Davaine.) "The sheep constantly fed in the sheep-folds were generally preserved." (Grognier, cited from Neumann.)

'09–'10. Fairbarn, under the nom de plume of "Lammermuir Farmer," lost 800 out of 2,000. (Treatise on the Cheviot and Black Faced Sheep.)

'12. Disease prevailed in the Midi (France) and especially in the departments of the Rhone, Herault and Gard; 300,000 sheep perished in Arles territory, and 90,000 in the district of Nîmes and Montpellier. (Davaine.)

1810 and 1811 were very wet years. (D Arboval.)

'16–'17. Bad years in England. (Simonds.)

'16–'17. Distomatosis caused such destruction in France that the Government appointed a special commission to study the malady.

'16–'17. Bad years for Germany. (Leuckart.)

'20. Severe epizoötic in environments of Beziers. (Davaine.)

'24. Distomatosis very destructive in England. Mr. J. Cramp lost £3,000 ($15,000) worth of sheep. (Simonds.)

'29–'30. Caused destruction in the majority of the localities in the department of the Meuse and neighboring departments. In the vicinity of Montmédy, out of 24,000–25,000 cattle, 5,000 perished; more than half of the sheep perished. Some localities lost 2,000 head of cattle and 1,500–1,800 sheep. Didry, 1832. (Davaine.)

In the vicinity of Verdun, of 20,000–21,000 cattle, 2,200

perished; of 50,000 sheep about 20,000 died. (Mangin, 1834, quoted from Neumann.)

'30–'31. Supposed to be the greatest outbreak England has experienced.

"Evidence of this immense destruction was given by various witnesses before the Parliamentary Committee, and it was satisfactorily ascertained that in 1833, two years afterwards, there were 5,000 sheep on every market day in Smithfield less than what used to be the average number, and 20,000 less than usual at Weyhill Fair." (Simonds.)

More than 2,000,000 sheep perished, representing a loss of £4,000,000 ($20,000,000 ! ! !) (Edin. Vet. Rev., 1861, after Leuckart.)

'34. Rot in Egypt. Evidently not an epizoötic. (The Veterinarian, 1834.)

'45. Spinola observed distomatosis in Poland (Polonge) and Russia, in conjunction with "la peste bovine." (D'Arboval.)

'51–'52. Epizoötic in Wurtemburg. (D'Arboval.)

'53–'54. England. Many thousands of sheep were swept away. (Simonds.)

France. Distomatosis reigned again in the greater part of France, especially in the departments of the centre; in Berry, Gatinais, and Lologne, stockowners lost from one to three-fourths of their stock. (After Delafond, quoted from Davaine.)

Germany. In the single district of Liegnitz, hundreds of thousands of sheep perished (D'Arboval.)

'60–'61. "The outbreak, which took place in the autumn and winter of 1860, proved as serious as that of 1830–31. Speaking in general terms, it may be affirmed that all the western and southern counties of England, together with several of the eastern and midland, then suffered to a ruinous extent. As in former years, so in this, the attacks of the disease were due to an excess and long continuance of wet weather. Eighteen hundred and sixty will be long remembered by agriculturalists, not only as producing the rot among sheep, but likewise for its baneful effects on the root-crop, and on the hay and corn harvests.

"We are acquainted with several instances, in the immediate neighborhood of Harrow-on-the-Hill, where the losses of sheep amounted from 600 to 700 in a flock. . . . In the winter of 1860–61, some persons lost all their sheep, and one farmer in particular, who had purchased between 800 and 900

Welsh ewes, had not more than 40 or 50 which escaped. A similar fatality attended the progress of the disease in other districts. In many parishes in Devonshire, where we investigated the malady, and of which Bridgerule may be taken as an example, five-sixths of the sheep perished, or were sold for a few shillings each for slaughtering, to the detriment of the health of the poorer classes. The Rev. S. M. Kingdon, the then resident minister at Bridgerule, reported to the author, that on October 1st, 1860, 492 sheep were existing in the parish as the joint property of several small farmers; and that by the end of the month, 410 of them had either died or been sold at a price very little above the value of their skins. In the instance thus particularized, the losses occurred among the stock of small occupiers, the ill consequences were greatly added to by their *young cattle* being found to be affected with flukes to such an extent as seriously to injure their health later on in the year.

" In Sussex and in several parts of Surrey the fatality was equally great. In the neighborhood of Eastbourne, a flock of about 600 Southdown ewes of great value was completely destroyed. Numerous cases of a similar kind might be named, but enough has been said to show not only the extent of the disease, but that sheep of every description, and placed under different systems of management, in like manner succumbed to the rot. It is much to be regretted that means did not exist whereby the total loss could be ascertained. People are left in doubt as to the amount of food of which they were deprived in one year by this disease alone, and of the efforts which had to be made to replace the loss. The time, we predict, cannot be far distant when agriculturalists will be convinced not only of the propriety but of the positive necessity of making accurate returns of the annual *losses* they sustain among their stock, instead of simply deploring them among themselves. Elsewhere, we have drawn attention to this important subject, upon which very much might now be said, if it were not somewhat unsuited to an essay of this kind.

" In Ireland, however, in consequence of the excessive rainfall of 1862, the losses from rot during the winter of that year were enormous. Professor Ferguson, in reporting on the disease to the Irish Government in February, 1863, says: ' never in the memory of the present generation has there been in this country such mortality among the ovine tribe as there is at present, and has been for the last few months, from what is generally designated rot.' To decide with any degree of accuracy the extent of the fluke malady existing at present, and that which existed during the past year, would be an impossibility.

"It, however, is my opinion that upwards of 60 per cent. of the sheep in the island are at present unsound, though not all to a fatal extent. . . . In 1861, there were 3,556,050 sheep in Ireland. In 1862, last year, at nearly the same period, when rot had not set in on its work of destruction, there was a deficiency of 100,163 from that number. That deficiency was incalculably increased during the remainder of this year. There will be a still far greater amount of deficiency this year (1863), an amount which I believe will be found greater than ever previously occurred in any year within the memory of the present generation, and certainly within the period of the Government collection of agricultural statistics." (Simonds '80.)

'72. Serious epizoötic in the greater part of Germany. (D'Arboval.)

'72–'73. The great majority of sheep slaughtered at Strasburg during this winter had flukes in their liver. (Zündel.)

'72–'74. Bassi reports epizoötic among the deer of the Royal Park near Turin, Italy. This epizoötic was due to *F. magna.*

'76. Slavonia lost 40 per cent. of its cattle from this disease. (Neumann.)

'79. England lost about 3,000,000 sheep. (Fleming's Neumann.)

'80. England's loss estimated at 3,000,000. Youatt estimates the annual loss in England at 1,000,000 sheep. In Australia the disease is even more constant than in England. (Fleming's Neumann.)

'82. Wernicke reports an epizoötic of this year in southern Buenos Ayres, in which at least 1,000,000 sheep were destroyed.

'86. During the first 8 months of this year more than 100,000 sheep were destroyed by flukes in the district of Tandil. (Wernicke.)

'89. Dinwiddie (vide infra) reports that for years the livers of cattle in Arkansas have been unfit for use. Mortality not given. (*F. magna.*)

'90. Francis investigated an outbreak in Texas where the loss ran up into hundreds of cattle. (Curtice.) *F. hepatica* or *F. magna?*

'91. Fascioliasis prevalent among the dairy cows of certain districts in California. (Curtice.) *F. magna.*

'91. "It is not generally known that the Trematoda bear an economic importance of the first magnitude to the live stock interest of Texas. . . . It is now three years since the investigations here recorded were begun. At first, the occurrence of enzoötics of 'Trematodism' were regarded as local, but on investigations it was found that these parasites cause serious damage almost every year." . . . (Francis.) Unfortunately, statistics are not given. *F. hepatica* and *F. magna.*

'91. A very severe year for Australia. One farmer reports a loss of 10,000 sheep. (Live Stock Journal, Oct. 30, 1891. After Fleming.)

'92. Lutz states that the liver-fluke has been recognized for some years in the Sandwich Islands, especially on Oahu. Only recently were its evil effects recognized. At time of writing, nearly the entire stock of cattle in some localities had been destroyed, while in other localities the disease was general but less severe.

---

In connection with these epizoötics it is interesting to note the ancient ideas which prevailed in regard to them. Zündel refers to the matter as follows :

"Formerly, when an epizoötic attacked the live stock, people were wont to attribute the malady to some supernatural influence, and often considered it a punishment from heaven. With this belief in some occult cause, the proprietor did nothing, or almost nothing, to combat the scourge ; people submitted to the will of God, and at the most they tried to appease the wrath of the Almighty by prayers and acts of devotion ; they tried to conjure the influence of the evil spirit—if they admitted that the epizoötic was the work of this one, by charms, by incantations, and even by exorcisms.

"A portion of this superstition, of this fatalism, has been preserved to our day, and often now, when an epizoötic breaks out in the stables of our cultivators, the latter submit humbly to their lot, not seeking the cause of the evil, not attempting to arrest its progress by means which their good sense should dictate to them. . . . . ."

Another liver-fluke, which has been known for some years, is a much smaller and less harmful one, the lancet-fluke *Distoma* (*Dicrocœlium*) *lanceolatum* Mehlis. We have not been able to find any specimens of this species in this country, although Leidy* states that it is recorded as frequent in several of the Western States. The parasite is quite common in sheep and cattle in certain parts of Europe.

In the early part of this century it was shown that dogs and cats harbor liver-flukes, and according to the more recent researches of †Max Braun, three species must be recognized, *i.e.*, *D.* (*Dicrocœlium*) *truncatum* (the truncated fluke), *D.* (*D.*) *felineum*, and *D.* (*D.*) *albidum*. All three of these flukes have been recorded in Europe, but thus far none of them have been found in this country. We have, however, found a new species of fluke in cats of New York, Maryland and the District of Columbia, which we will shortly describe. (See Notes on Parasites—21.) These parasites bring about a fluke disease in dogs and cats similar to, but less severe than the corresponding disease in sheep.

In 1847 Dr. Jackson showed that elephants also were infested with liver-flukes, and according to later accounts the worm which Jackson found in an elephant in Boston causes serious losses among the elephants in India. This worm has been named *F. Jacksoni*.

Some years later (1856) Cobbold showed that a somewhat similar parasite (*F. gigantea*) is found in giraffes, while in 1875 Bassi recorded another liver-fluke, which he named *D. magnum*, as the cause of a serious epizoötic among the deer of the Royal Park, near Turin, Italy, during the years 1872–1874. Bassi's fluke was then supposed by most authors to be identical with the common fluke, *F. hepatica*.

Within recent years it has been noticed that the cattle in certain parts of this country are seriously infested with liver-flukes, and it has been determined that while *F. hepatica* is by no means uncommon in certain districts, the more common form is identical with the form discovered by Bassi, a worm to which the name *Fasciola magna* should be given. Dinwiddie has found that in some counties of Arkansas practically all the cattle harbor these parasites, and he has also found them in cattle from Indian Territory. Francis reports this form as very common in Texan cattle, a report which Dr. Melvin and myself can corroborate from our examinations of Texan cattle at the Chicago abattoirs.

As this parasite is so frequent in certain districts, and as it is so much larger than the common liver-fluke, *F. hepatica* that it

*Proc. Phila. Acad. Sc. 1856.

†C. f. B. u. P. XIV. pp. 381–392, 422–428. 1893.

must be looked upon as even a more dangerous parasite than that form, it is important for us to learn all we can of its history, structure and life-cycle, in order to be prepared to combat the disease it causes ; at the same time, while studying one fluke, or one fluke-disease, it will be well to draw closely allied forms into comparison. As *Fasciola magna* has been described but very superficially heretofore, it became necessary to study the parasite more carefully in order to determine its exact relations to *F. hepatica*, and to obtain definite specific characters which would more easily distinguish it from that species, and it was also necessary to search the American literature for accounts of disease which could be referred to this parasite. This study of the literature and the anatomy—a necessary prelude to writing an intelligible specific diagnosis—resulted in the writing of this paper. The facts brought out in the paper, *i. e.*, that the anatomy of *Fasciola magna* is so very similar to the anatomy of *F. hepatica*, points to the probability that the life-history of the two forms will prove to be very nearly the same.

Before passing to the anatomy of *F. magna*, it may be well to consider for a moment how many different forms of liver-flukes are found in man and the larger domesticated animals. The following table gives a concise idea of our present knowledge of distribution of these forms in man, cattle, sheep, goats, horses, asses, hogs, dogs, rabbits, cats, elephants, giraffes and camels.

| Parasites. | F. hepatica | F. magna | F. Jacksoni | F. gigantea | D. lanceolatum | D. sinense | D. conjunctum | D. truncatum | D. felineum | D. albidum | D. sp. nov. S. & H. MS. | Amphistoma explanatum |
|---|---|---|---|---|---|---|---|---|---|---|---|---|
| Host | | | | | | | | | | | | |
| Man | x | | | | x | x | x | | | | | |
| Cattle | x | x | | | x | | | | | | | |
| Zebu | | | | | | | | | | | | x |
| Sheep | x | | | | x | | | | | | | |
| Goats | x | | | | | | | | | | | |
| Horses | x | | | | | | | | | | | |
| Mules | | | | | | | | | | | | |
| Asses | x | | | | | | | | | | | |
| Hogs | x | | | | x | | | | | | | |
| Rabbits | x | | | | x | | | | | | | |
| Dogs | | | | | † | | x | x | x | | | |
| Cats | x | | | | † | x | | x | x | x | x | |
| Elephants | | | x | | | | | | | | | |
| Giraffes | | | | x | | | | | | | | |
| Camels | x | | | | | | | | | | | |

x Present.
† The form described as *D. lanceolatum* in dogs and cats is *D. felineum*.

Of these parasites, the first four flukes form a very natural group of closely allied species, showing certain characters in common which justify us in separating them zoologically from the other flukes (cf. infra). These flukes are also the largest of the flukes, and at the same time the most dangerous. They are the only ones which will be considered in this paper.

The next seven flukes are much smaller, and do not produce such serious effects upon their hosts. They too present certain characters in common, some of which are entirely different from those of the genus *Fasciola*. They are placed in the sub-genus *Dicrocœlium* of the genus *Distoma*.

As members of the genus *Distoma*, they present the following characters* in common:

1. The animals are hermaphrodites;
2. The intestinal cæca are simple;
3. The ovary is round or lobate;
4. The testicles are round, lobate, or slightly branched;
5. The vitellogene gland is marginal, but not so profusely developed as that of *Fasciola*.

As members of the sub-genus *Dicrocœlium*, they present the following characters:

1. The intestinal cæca are long and simple;
2. The oral sucker is without spines or lobes;
3. An œsophagus is present;
4. The acetabulum is sessile;
5. The genital pore is immediately anterior to the acetabulum.

The sub-genus *Dicrocœlium* is subdivided into two sections, according to the relative position of the testicles to the uterus. Of the Distomes here under discussion, *D. lanceolatum* belongs to the first section of the sub-genus, and the remaining flukes to the second section:

First Section. The genital glands lie anterior to the uterus; a cirrus is present: *D. lanceolatum.*

Second Section. The genital glands lie posterior to the uterus; the testicles are never exactly side by side, but one is regularly more or less posterior to the other. The oral sucker is

---

*In this and the following tables the characters have been taken which are common to the flukes under discussion, but it must not be assumed that all of the characters mentioned will necessarily be found in all the other members of the respective groups. In other words, the characters given are not proposed as revised diagnoses of the different groups.

followed by a pharynx; there is no cirrus present; the vitellogene glands never extend posterior to the testicles.

In this section the flukes here under consideration naturally fall into two sub-sections:

*a.* Testicles round, oval or somewhat lobate; end portion of the excretory canal has a sigmoid course between the testicles: *D. conjunctum*, *D. felineum*, *D. albidum*, *D. sp. n.*, and probably *D. truncatum.*

*b.* Testicles branched; end portion of the excretory canal runs nearly straight and lies dorsally of the testicles: *D. sinense.*

The last fluke (*A. explanatum*) mentioned in the above list is found in India. The acetabulum is posterior.

In the present paper we will first study the large American fluke (*F. magna*), comparing it with the "common fluke," *F. hepatica;* from this we will pass to account of *F. Jacksoni* and *F. gigantea*, and a review of the question as to the generic name *Fasciola.* The "common fluke" (*F. hepatica*) has been so thoroughly studied by other authors that it is not necessary to go into an account of the anatomy any further than can be done in comparing it with the other forms.

The material at my disposal consists of a large number of specimens collected as follows :

1. Eight specimens of *F. magna* from *Cervus dama*, forwarded by Prof. Sonsino of Pisa, Italy.
2. Several specimens collected by Dr. Curtice at Colorado Springs (Hassall's type-specimens of *Fasciola carnosa* seu *americana*).
3. Several specimens collected by Dr. Francis in Texas, and sent to the late Prof. Leidy. These were determined by Leidy as *D. crassum* (Co-types of Francis' *Distomum texanicum*).
4. A number of specimens sent to this Bureau by Dr. Dinwiddie from Arkansas.
5. A number of specimens sent from Chicago by Dr. Melvin.
6. Eight specimens found by Dr. Hassall in a Texan steer slaughtered at Washington, D. C., March, 1893.
7. A number of specimens collected by myself in Chicago in August, 1893.

In the Chicago abattoirs about one-fourth of the Texan cattle I examined during August, 1893, had flukes (*F. magna*, *F. hepatica*, or both) in their livers. The parasites were found both in the bile-ducts and in the parenchyma. In most of the cases they were contained in cysts (cf. Fig. 1, Pl. 1), two or three to ten or twelve in each cyst. These cysts varied in size from a walnut to an apple, and did not seem to be confined to any particular portion of the liver, but were found near the periphery, or near the center, on the convex surface, or on the concave surface, etc. No statistics were taken, however, to determine their relative occurrence in the different lobes. The cysts naturally produced an irregularity of the surface of the liver, so that they were easily detected. They were generally very hard, there being in many cases a calcareous shell surrounding the cavity. The cavity of the cyst contained, besides the flukes, a dirty greenish to brown or black substance of mushy or gritty consistence, in which were found numerous fluke eggs, blood corpuscles and a mass of gritty material. The livers containing these parasites were darker in color than the normal liver.

### I. Fasciola magna (Bassi 1875).

PLATES I AND II AND FIGURES A–G.

SYNONYMY: 1875, *Distomum magnum* Bassi;
1882, *Distoma grande* Perroncito, Italian (nec Rud., 1819, Latin);
1887, *D. hepaticum* ex. p. Curtice;
1889, *Fasciola hepatica* ex. p. Dinwiddie;
1891, *F. carnosa* Hassall;
1891, *F. americana* Hassall;
1891, *D. texanicum* Francis;
1891, *D. crassum* ex. p. Leidy;
1892, *Cladocœlium giganteum* ex. p. Stossich;
1894, *F. magna* (Bassi, 1875) Stiles.

COMMON NAMES: English—The Large American Fluke;
German—Der grosse amerikanische Leberegel;
Italian—Distoma grande, D. magno.

HOSTS: Cattle (*Bos taurus*), reported by Curtice, Francis, Dinwiddie, Hassall and Stiles;
Blue Bull (*Bosephalus tragocamelus*), Bassi;
Sambur (*Cervus* (*Rusa*) *unicolor*), Bassi;
Elk or Wapiti (*Cervus canadensis*), Bassi;
Fallow Deer (*Cervus* (*Dama*) *dama*), Bassi, Sonsino;
Stag (*Cervus elaphus*), Bassi;
Virginia Deer or Red Deer (*Cariacus virginianus*), Leidy.

GEOGRAPHICAL DISTRIBUTION—*North America:* Texas (Francis); Arkansas (Dinwiddie, Hassall and Stiles); Indian

Territory (Dinwiddie); California (Curtice); Iowa (H. Osborn); Illinois, Union Stock Yards of Chicago, in Texan cattle (Stiles, Judd, Norgaard, et al.); Adirondacks, N. Y. (after Leidy). *Europe:* Italy (Bassi and Sonsino).

LITERATURE 1875–1894.

BASSI—

'75.—Sulla cachessia itteroverminosa o marciaia dei Cervi, causata dal Distomum magnum; Il Medico Veterin. Torino, pp. 497–513, Tav. I–III.

BRAUN—

'93.—Vermes: Bronn's Klassen und Ordnungen des Thierreichs.

CURTICE, Cooper—

'87.—Distoma in Livers and Lungs of Cattle; American Veterinary Review, pp. 390–392.

'91.—Flukes in Cattle and Sheep; Pacific Rural Press, January 24.

'92.—Parasites; JOURNAL OF COMPARATIVE MED. AND VET. ARCH., p. 223.

DINWIDDIE, R. R.—

'89.—Veterinarians' Report. Second Annual Report of the Arkansas Agricultural Experiment Station, pp. 109–119. 1 Fig.

'92.—Some parasitic affections of Cattle; JOURNAL OF COMPARATIVE MED. AND VET. ARCH., pp. 342–343.

FRANCIS, M.—

'91.—Liver Flukes; Texas Agricultural Station Bulletin. No. 18, October, pp. 135–136, 5 Figs. and map. Ref. (Stiles) Centralblatt für Bakteriologie und Parasitenkunde, 1892, p. 608. Vol. XI.

'92.—Notes on Parasites; JOURNAL OF COMPARATIVE MED. AND VET. ARCH., p. 426.

HASSALL, Albert—

'91A.—A new species of Trematode infesting Cattle (*F. carnosa*); American Veterinary Review, pp. 289–290. 1 Fig. — Ref. (Stiles) Centralblatt für Bakteriologie und Parasitenkunde, Vol. X, p. 464. . . . Rev. (Stiles) American Naturalist, Jan., 1892, p. 65. . . . Rev. (Bell) Journ. Royal Micr. Soc., 1892.

'91B.—*Fasciola americana;* American Veterinary Review, p. 359.

'94.—List of the Chief Epizoötics of Fascioliasis (Distomatosis); JOURNAL OF COMPARATIVE MED. AND VET. ARCH., pp. 162-167.

LEIDY, Joseph—
'91.—Notes on Entozoa; Proc. Acad. Nat. Sci. Phila., p. 234–236. *D. crassum.*

LEUCKART, R.—
'79–'94.—Die Parasiten des Menschen, 2te. Aufl., l. p. 298.
'92.—Ueber den grossen amerikanischen Leberegel; Centralblatt für Bakteriologie und Parasitenkunde, XI, pp. 797–799.

OSBORN, Herbert, and YOUNG, G. V.—
'90.—American Field, March 1, p. 199.

PERRONCITO, E.—
'82.—I Parassiti dell' Uomo è degli Animali utili, p. 284. Milano.
'86.—Trattato teorico-pratico sulle Malattie più comuni degli Animali domestici, etc., p. 249. Torino.

RAILLIET—
['93–'94.]—Traité de zoölogie médicale et agricole, 2.éd. Paris (although the first part of this work—upon which I am laying great expectations as a scientific treatise bringing the subject of parasitism up to date—has appeared in France, it has not yet reached this country).

SONSINO, Prospero—
['90.]—Studie notizie elmintologiche; Proc. verb. d. sociéta Toscana di scienze natur. 4 maggio, 16 pgs. . . . Ref. (Braun) C. f. B. u. P., VIII, p. 309.

STILES, [Charles] Wardell—
'92A.—Notes on Parasites—7: A word in regard to Dr. Francis' *Distomum Texanicum;* American Veterinary Review, pp. 732–733. Abstract: JOURNAL COMPARATIVE MED. AND VET. ARCH., 1892, p. 148.
'92B.—Notes on Parasites—11: *Distoma magnum* Bassi, 1875; JOURNAL COMPARATIVE MED. AND VET. ARCH., pp. 464–466.
'94.—The Anatomy of the Large American Fluke (*Fasciola magna*), and a Comparison with other Species of the Genus *Fasciola*, s. st. Containing also a list of Chief Epizoötics of Fascioliasis (Distomatosis) and a Bibliography of *F. hepatica* by Albert Hassall (the present paper); JOURNAL OF COMPARATIVE MED. AND VET. ARCH.

STOSSICH, M.—

'92.—I Distomi dei Mammiferi; Programma della civica Scuola Reale superiore. Trieste.

HISTORICAL REVIEW.

BASSI ('75) had occasion to observe an epizoötic among the deer of the Royal Park near Turin, Italy, in which the symptoms shown by the animals were essentially the same as those shown by sheep infested with *Fasciola hepatica.* In the post-mortem examinations it was found that the deer were heavily infested with flukes, which differed somewhat from *F. hepatica*, and which Bassi then described as a new species, *Distomum magnum.* He was inclined to believe that the parasites were introduced in the Park by the Wapiti (*Cervus canadensis*). The effects of the entozoa became marked in 1872, and increased notably in 1873 and 1874, the disease being at its highest during the winter and spring. Bassi describes the pathological changes produced by the worm, but his description of the parasite itself is so unsatisfactory that LEUCKART ('79) and others did not hesitate to pronounce the fluke identical with *F. hepatica.*

PERRONCITO ('82) accepts Bassi's species, evidently taking his diagnosis from Bassi's article. His diagnosis is headed "Distoma grande. *Distoma magno* (*Distoma magnum*, Bassi)."

As Perroncito has headed the other species of flukes with the Italian names (cf. l.c. p. 273 Distoma epatico, p. 276 D. lanceolato, etc.) giving the Latin scientific name in brackets, I suppose that he intended to use the term *grande* as an Italian word, and not to propose it as a new Latin name for this species, especially as the specific term *D. grande* was already preoccupied by Rudolphi (1819) for a fluke in a South American Spoonbill, *Ajaja ajaja* (L) (*Platalea ajaja.*) As *grande* is, however, both a Latin and an Italian word, and as this point might possibly lead to slight confusion in indexing species, I have included the term *D. grande* Perroncito, 1882, among the synonyms.

CURTICE ('87) records three cases of pulmonary distomatosis in Kansas cattle. He considered the parasites identical with *F. hepatica*, but has since stated (personal conversation) that they were in reality *F. magna.* He also cites an article in the New York "Tribune" of 1870-71, by J. H. Batty, recording liver-flukes in Virginia deer. Curtice adds: "The species is undetermined, but closely resembles *D. hepaticum*, which it probably was." Curtice also cites A. J. Murray (Veterinary Review, 1882), as recording three cases of pulmonary distomatosis in Texan cattle.

DINWIDDIE ('89) had occasion to examine a large number of cattle affected with these flukes. He stated that for years the livers of cattle from certain districts had been unfit for use. As the district covered by this disease fell within the permanently infected area of Texas cattle fever, a number of persons had supposed that the changes brought about by the liver-fluke were due to Texas fever. Post-mortem account of a steer, four years old, is given as follows:—

"Apparently in good health and fair butchering condition. The 'fat caul' seen on first opening the abdomen as a large sheet, was dotted with black spots and streaks. Lymphatic glands on the concave surface of the liver were much swollen and black in color. The liver itself was enlarged and darkened on the surface, with a number of prominent elevations, some appearing like blisters, and some more or less solid, and varying greatly in size. A longitudinal section showed the presence of many cavities, some containing a dark fluid in which were floating granules and shreds of tissue. One very large cavity, about two inches in diameter, with irregular yellowish colored walls, besides the dark colored fluid above mentioned, contained also two flat, leaf-like bodies about one inch in length and slightly less in breadth. They were fished out and recognized as 'Flukes.' . . . More of these were obtained from other cavities. Several other cavities contained solid, greenish, yellow gritty matter, and no parasites. A section made through the liver in any direction, cut through one or more of these cysts. They were situated near the surface of the organ or in its substance indiscriminately. Those that contained the 'Fluke' were usually of medium or smaller size, and the parasite was found folded or curled upon itself longitudinally and surrounded by fluid. . . . The shreds of tissue found in those cysts, which did not contain the living parasites, were shown by microscopic examination to be the débris of dead and partly decomposed Flukes.

"Such were the gross appearance of the livers of at least three-fourths of the cattle slaughtered during the spring and summer at this place, and of about ninety per cent. of all coming from certain ranges in St. Francis and Lee counties."

In August and September this condition was common, no living flukes being found.

Dinwiddie then gives a short description of the parasite, the life-history of *F. hepatica*, and practical measures to be adopted to prevent the disease. From the figure given, as well as from Dinwiddie's latter statements, it is evident that the parasite he found was *F. magna*, although he at that time called it *F. hepatica.*

As the summer advanced, Dinwiddie noticed that the flukes were less common in the livers, but that "cicatrices or scars, the evidence of former inflammatory actions," were found.

Osborne ('90) examined some "leeches" which had been found in a deer, and determined them as "liver-flukes." It seems probable that they belonged to this species.

Sonsino ('90) records this species from *Cervus dama* in Italy. He expresses the opinion that the form may be identical with *F. gigantea* Cobbold. [Original paper not at hand, I quote from Braun's review.] Sonsino records a specimen 10 cm. long.

An article appeared in the Pacific Rural Press, in which a farmer stated that a disease was prevalent among the dairy cows of a certain district. He opened one of them and found "thousands of parasites, resembling diminutive flat-fish." In reply to this letter Dr. Curtice ('91) stated that the parasites in question were liver-flukes, and more recently, in private conversation, he has told me that the species found in this case was identical with the species now under discussion. In the article referred to, Curtice states that Francis had the winter previous investigated an outbreak in Texas, where the loss ran up into hundreds of cattle.

Hassall ('91a) found several specimens of flukes (collected by Curtice) in the collection of this Bureau under the label *D. hepaticum.* He recognized that they were distinct from *F. hepatica* and gave a short description of them as *Fasciola carnosa* sp. n. Later ('91b) Hassall changed the name to *Fasciola americana,* since the specific name "*carnosa*" had already been given to another fluke (*Distoma carnosum* Rud. in *Dentex vulgaris*).

About one month later, Francis ('91) re-described the same parasite ; not being acquainted with Bassi's paper, and overlooking Hassall's two publications, he re-named the species *Distomum Texanicum.* Attention was called to this by Stiles in his review of Francis' paper, and by Leuckart ('92).

The largest flukes Francis found measured 73 mm. long. Usually 10–15 were found in a single liver, but upon one occasion 27 were found. They are described as wandering aimlessly around in any direction, "in channels which they had produced. . . . The majority are near the convex or outer surface of the liver. The channels they produce admit the little finger, and seem to heal or fill up soon after, leaving a red scar. Sometimes they perforate the surface of the liver, then suddenly turn back into the liver again. I think that they sometimes leave the liver and bore into adjoining tissues or organs, but I have not found them in other places than the liver."

LEIDY ('91) examined specimens sent to him by Francis, and also specimens from *Cariacus virginianus* (Adirondack Mountains, N. Y.), and from a calf from Hot Springs, Ark. He determined the forms as identical with *Distoma crassum*, which he had found in a Chinese boy. Not knowing how prevalent the large fluke really was in America, he thought it possible that the worms were introduced into this country by immigrants from China.

STILES ('92A) states that the parasite described by Francis as *Distomum texanicum* is identical with Hassall's *Fasciola americana* and probably identical with Bassi's *D. magnum.*

DINWIDDIE ('92) states that in four or five counties of Arkansas practically all the cattle harbor these liver-flukes, while in many other counties, as well as in cattle from Indian Territory, the disease is occasionally met with. The livers of these cattle are unfit for food. He has never found this particular form in sheep. Dinwiddie says that when he first found these parasites he doubted whether they were really identical with *F. hepatica,* although he at that time published them under that name.

FRANCIS ('92) in his second paper, adds nothing of any scientific nature, but discusses the question of priority. A reply to this paper may be found in a postscript to my Notes on Parasites —11.

LEUCKART ('92) examined a specimen of *Fasciola americana*, compared it with one of Bassi's original specimens of *D. magnum*, and pronounced the two specifically identical. He considers that *D. magnum* was introduced into Italy by **Cariacus virginianus.*

STILES ('92B), not knowing that Leuckart's paper was in press, examined eight specimens of Bassi's *D. magnum*, sent to him by Sonsino, and states that there is no doubt in regard to the identity of this form with the American species, *Fasciola americana* (*D. texanicum*).

STOSSICH ('92, p. 9) unites this species with *F. gigantea* under the term *Cladocœlium giganteum.*

Braun's statements ('93) are based upon Leuckart's article ('92).

*Specific name.*—I have already shown in another paper ('91) that the specific name *magna* should be retained for this species.

* Leuckart undoubtedly intended to refer to *Cervus canadensis*, the Wapiti.

(*To be continued.*)

